# Why Is PaO_2_ Not Enough? Arterial Oxygen Content as a Prognostic Indicator in COPD Patients

**DOI:** 10.5041/RMMJ.10573

**Published:** 2026-04-26

**Authors:** Stephany Ivonne Briones Alvarado, Walther Iván Girón Matute

**Affiliations:** 1 Department of Respiratory Medicine, Gregorio Marañón General University Hospital, Madrid, Spain; 2 Faculty of Medicine, Complutense University of Madrid, Madrid, Spain; 3 Gregorio Marañón Health Research Institute (IiSGM), Madrid, Spain

**Keywords:** COPD, mortality, oxygen

## Abstract

**Background:**

Chronic hypoxemia in patients with COPD is associated with increased morbidity and mortality. Although arterial partial pressure of oxygen (PaO_2_) is widely used, it does not adequately reflect systemic oxygen transport. Arterial oxygen content (CaO_2_) may provide a more comprehensive assessment.

**Objective:**

This study aimed to evaluate whether or not CaO_2_ is a better predictor of mortality than PaO_2_ in patients with COPD.

**Methods:**

This retrospective observational cohort study included 147 COPD patients aged ≥45 years. Patients were categorized according to CaO_2_ levels (low, normal, high). Mortality at 1, 3, and 5 years was analyzed. Statistical methods included ROC curves, Kaplan–Meier survival analysis, and Cox regression models.

**Results:**

A total of 66 deaths (45.2%) occurred in the study cohort. Mortality was highest in the low CaO_2_ group. The CaO_2_ demonstrated better predictive performance than PaO_2_ (AUC 0.73 versus 0.53, respectively). Low CaO_2_ was associated with a 2.5-fold increased risk of mortality. Despite improvements in PaO_2_ after long-term oxygen therapy, CaO_2_ did not significantly change.

**Conclusions:**

The CaO_2_ is a more informative marker of oxygen transport and mortality risk than PaO_2_ in COPD patients. It should be considered a complementary parameter in clinical assessment.

## BACKGROUND

Uncorrected chronic hypoxemia in patients with chronic obstructive pulmonary disease (COPD) is associated with complications such as pulmonary hypertension, secondary polycythemia, systemic inflammation, and skeletal muscle dysfunction, all of which contribute to impaired quality of life and increased morbidity and mortality.^1^,^2^ While arterial partial pressure of oxygen (PaO_2_) is a commonly used indicator of alveolar ventilation, it does not adequately reflect the oxygen transport capacity, as the majority of oxygen is carried bound to hemoglobin (Hb).^3^ Arterial oxygen content (CaO_2_) is defined as the total amount of oxygen carried in arterial blood and is calculated as follows: CaO_2_ = (1.34 × Hb × SatO_2_) + (0.003 × PaO_2_), where Hb is the hemoglobin concentration, SatO_2_ is the arterial oxygen saturation, and PaO_2_ is the arterial partial pressure of oxygen. While PaO_2_ reflects only dissolved oxygen, CaO_2_ integrates both oxygen bound to hemoglobin and dissolved oxygen and therefore better represents systemic oxygen transport.^3^,^4^

Accordingly, CaO_2,_ which depends on Hb concentration and saturation, provides a more comprehensive measure of systemic oxygen delivery.^3^,^4^ Anemia, even at mild levels, can significantly lower CaO_2_ and overwhelm compensatory mechanisms (e.g. increased cardiac output, oxygen extraction ratio) triggered by hypoxemia,^5^–^8^ compromising tissue oxygenation even with normal PaO_2_. Based on this physiological rationale, we hypothesized that CaO_2_ may serve as a more accurate predictor of mortality and response to long-term oxygen therapy (LTOT) in COPD patients, compared to PaO_2_ alone.

## MATERIALS AND METHODS

This was a retrospective observational cohort study to evaluate mortality risk in COPD patients based on their CaO_2_ and PaO_2_ values. The study was conducted in a single tertiary-care hospital. A subanalysis stratified patients into three categories according to CaO_2_ levels: low (<16 mL/dL), normal (16–20 mL/dL), and high (>20 mL/dL). The CaO_2_ categories were defined *a priori* based on previously published reference values.^5^,^6^ All patients were initially evaluated in the outpatient setting, where baseline arterial blood gas measurements were obtained under stable clinical conditions and used for the primary analyses. In patients who subsequently initiated LTOT, follow-up arterial blood gases were available in a subset of cases during later hospital admissions for COPD exacerbations. These measurements were obtained as part of routine clinical care prior to hospital discharge, once clinical stability had been achieved. The LTOT prescription was based on standard clinical criteria and was not determined by CaO_2_. Patients aged ≥45 years with a confirmed diagnosis of COPD, seen in medical consults between 2018 and 2024, were eligible.

Exclusion criteria included poor adherence to inhaled therapy, need for chronic mechanical ventilation, multi-organ failure, interstitial lung disease, pulmonary embolism, significant cardiovascular disease, active malignancy, chronic kidney disease (glomerular filtration <30 mL/min), Child–Pugh B/C cirrhosis, hemoglobin <9 g/dL, or other conditions limiting life expectancy to less than one year.

Poor adherence was defined as documentation in the medical record of non-compliance with the prescribed inhaled treatment or LTOT, as assessed by the pulmonologist responsible for the treatment.

Mortality was measured throughout the study period, reporting survival at 1, 3, and 5 years. Mortality data were obtained from the hospital electronic medical record system, which is integrated with the regional health information system and linked to the national mortality registry. This allowed identification of deaths occurring both within and outside the hospital setting. Therefore, vital status was available for the entire cohort, and no significant loss to follow-up occurred during the study period.

Data were extracted from electronic medical records. Values for PaO_2_, Hb, and CaO_2_ were obtained from arterial blood gas analyses. Baseline arterial blood gas measurements were obtained while patients were breathing room air. In patients who subsequently initiated LTOT, a second arterial blood gas measurement was available in a subset of cases during later hospital admissions for COPD exacerbations. These follow-up measurements were obtained as part of routine clinical evaluation prior to hospital discharge, once patients had reached clinical stability and while receiving their prescribed LTOT. Arterial blood gases were analyzed using the GEM Premier 5000 system (Werfen, Bedford, MA, USA). Descriptive statistics included means, standard deviations (SD), frequencies, and percentages. Comparative analyses used Student’s *t*-test, ANOVA, and Fisher’s exact test. Diagnostic performance for mortality was assessed with ROC curves, with cutoffs defined using the Youden index. Kaplan–Meier survival analysis was used to estimate survival probabilities across CaO_2_ categories.

Time-to-event analyses were primarily performed using Cox proportional hazards regression models. Logistic regression analyses were conducted as complementary analyses to estimate odds ratios for mortality across CaO_2_ categories. Baseline characteristics were summarized by CaO_2_ group. Between-group imbalance was assessed using standardized mean differences (SMD), in accordance with recommendations for observational cohort studies. Hospitalization status (outpatient versus inpatient) was included as a covariate in the multivariable analyses. Statistical analysis was performed using SPSS (v25.0.0.0, Armonk, NY, USA); statistical significance was set at *P*≤0.05.

## RESULTS

After applying the inclusion and exclusion criteria, a total of 147 patients were included in the study. Baseline sociodemographic, clinical, and functional characteristics according to CaO_2_ category are shown in Table 1 and Table 2.

**Table 1:** Baseline Sociodemographic and Clinical Characteristics of the Study Population (*n*=147).

Characteristic	Low (*n*=46)	Normal (*n*=92)	High (*n*=9)	SMD Low vs Normal	SMD High vs Normal
Age (years)	74.8±11.66	69.1±12	68.6±11.25	0.48	−0.04
BMI	22.5±2.1	25.4±3.9	23.2±2.7	−0.85	−0.58
Pack-years	47.7±19.91	58.3±28.17	37.1±14.76	−0.41	−0.78
Years of COPD	12±1.97	11±2.3	9±2.1	0.46	−0.88
BODEx	3±1.1	3±1.2	2±1.1	0.00	−0.84
Gender (male)	37 (80.4%)	66 (71.7%)	9 (100%)	0.19	1.00
LTOT	21 (46.7%)	37 (40.2%)	0 (0%)	0.13	−1.14
Chronic heart failure	17 (37.8%)	20 (21.7%)	0 (0%)	0.38	−0.74
Hypertension	32 (68.9%)	50 (54.3%)	2 (22.2%)	0.30	−0.78
Chronic kidney disease	7 (15.6%)	8 (8.7%)	0 (0%)	0.28	−0.44
Diabetes	14 (31.1%)	25 (27.2%)	2 (22.2%)	0.09	−0.13
Dyslipidemia	19 (42.2%)	49 (53.3%)	3 (33.3%)	−0.22	−0.48
Obstructive sleep apnea	7 (15.6%)	16 (17.4%)	2 (22.2%)	−0.06	0.13

Values are expressed as means±SD or as numbers (%).

BMI, body mass index; BODEx, BMI, airway obstruction, dyspnea, and exacerbations; COPD, chronic obstructive pulmonary disease; LTOT, long-term oxygen therapy; SMD, standardized mean difference (low vs normal, high vs normal).

**Table 2:** Spirometry Data, Number of Exacerbations, Number of Hospital Admissions, and Hb Values (*n*=147).

Parameter	Low CaO_2_ (*n*=46)	Normal CaO_2_ (*n*=92)	High CaO_2_ (*n*=9)	*P* Value
Hb (g/dL)	11.1±1.98	13.1±1.20	17.4±2.90	0.01
FEV1 (mL)	1136.4±420.46	1200.2±471.98	1504.6±610.91	0.01
FEV1 (%)	47.8±16.77	50.9±17.94	51.6±18.44	0.327
FVC (mL)	2266.8±678.37	2322.9±738.00	3202.0±886.91	0.002
FVC (%)	79.1±20.91	76.2±21.33	85.8±20.41	0.366
FEV1/FVC (%)	48.8±10.95	49.9±11.27	47.5±12.93	0.755
Flare-ups last 12 months	2.1±2.70	1.9±2.85	0.8±0.97	0.40
Hospitalized exacerbations	1.5±2.65	1.1±1.72	0.7±0.87	0.452

Values are expressed as means±SD or as numbers (%).

Low CaO_2_, <16 mL/dL; Normal CaO_2_, 16–20 mL/dL; High CaO_2_, >20 mL/dL.

Hb, hemoglobin; FEV1, forced expiratory volume in 1 second; FVC, forced vital capacity.

During the study period, 66 deaths (45.2%) occurred among the 147 patients in the cohort. Mortality differed across CaO_2_ categories, with the highest mortality observed in the low CaO_2_ group and the lowest in the high CaO_2_ group (Table 3).

**Table 3:** Mortality According to Arterial Oxygen Content (CaO_2_) Category.

CaO_2_ Category	Patients, *n*	Deaths, *n*	Mortality, %
Low (<16 mL/dL)	46	33	71.7
Normal (16–20 mL/dL)	92	31	33.7
High (>20 mL/dL)	9	2	22.2
Total	147	66	45.2

**Figure 1:**
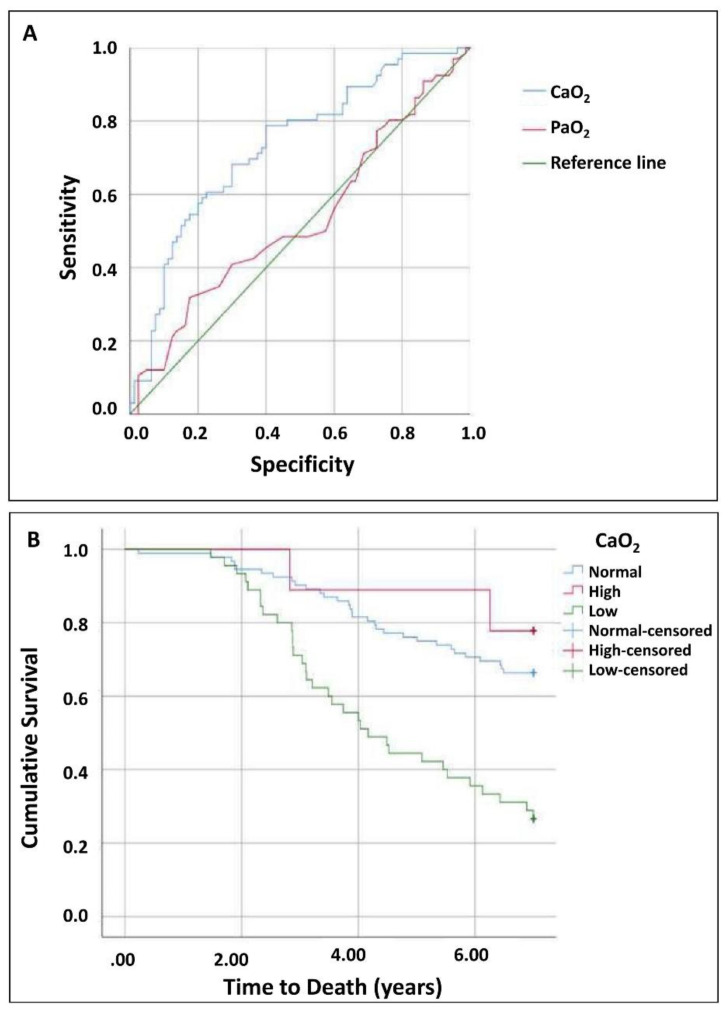
Discriminatory Ability and Survival According to CaO_2_. **A:** ROC curves comparing the predictive ability of arterial oxygen content (CaO_2_) and arterial partial pressure of oxygen (PaO_2_) to predict mortality. CaO_2_ showed greater discriminatory ability, evidenced by a higher area under the curve (AUC) compared to PaO_2_. **B:** Kaplan-Meier survival curves stratified according to CaO_2_ levels (high, normal, and low). Patients with low CaO_2_ had lower cumulative survival over time, suggesting a significant association between reduced CaO_2_ and mortality.

A strong correlation was observed between Hb and CaO_2_ (*r*=0.858; *P*<0.01), while the correlation between PaO_2_ and CaO_2_ was weak (*r*=0.144; *P*<0.001). Older age was associated with an increased risk of low CaO_2_ (OR 1.1; 95% CI 1.017–1.088; *P*=0.030), increasing by approximately 60% per decade (OR 1.6; 95% CI 1.18–2.31). Chronic heart failure (CHF) was also associated with low CaO_2_ (OR 2.3; 95% CI 1.082–5.083; *P*=0.031), with an even higher risk in patients with heart failure and reduced ejection fraction (HFrEF) (OR 3.5; 95% CI 1.63–7.69; *P*=0.010), in contrast to those with heart failure and preserved ejection fraction (HFpEF) (OR 1.04; 95% CI 0.48–2.25; *P*=0.92). Arterial oxygen content (CaO_2_) showed better predictive ability for mortality than PaO_2_ (Figure 1), with an AUC of 0.73 (*P*<0.01; 95% CI 0.65–0.81) versus 0.53 for PaO_2_ (*P*=0.050; 95% CI 0.44–0.63), indicating that PaO_2_ was not a useful predictor in this cohort, whereas CaO_2_ demonstrated moderate predictive accuracy.

The optimal cutoff point for CaO_2_ was ≤17.4 mL/dL, with a sensitivity of 79% (95% CI 67.9%–87.1%) and a specificity of 40% (95% CI 29.9%–50.9%). The positive predictive value (PPV) was 52.5% (95% CI 42.8%–61.9%), and the negative predictive value (NPV) was 69.6% (95% CI 55.2%–80.9%). In comparison, the cutoff point for PaO_2_ was ≤53.5 mmHg, with a sensitivity of 31%, specificity of 18%, PPV of 24.1%, and NPV of 23.3%. Patients with low CaO_2_ had a 2.5-fold higher risk of mortality (95% CI 1.516–4.271; *P*<0.01), adjusted for age, smoking, lung function, CHF, and years of COPD progression. These variables were selected for their potential role as confounding factors in the relationship between CaO_2_ and mortality. High CaO_2_ was associated with reduced mortality risk (OR 0.64; 95% CI 0.42–0.97; *P*=0.04). Survival at 1, 3, and 5 years was 93.3%, 71.1%, and 42.2% in the low CaO_2_ group; 98.9%, 90.2%, and 76.1% in the normal CaO_2_ group; and 100%, 97.9%, and 89% in the high CaO_2_ group, respectively. All patients included in the cohort were initially evaluated in the outpatient setting, and baseline arterial blood gases were obtained during stable clinical conditions. Therefore, the primary analyses were based on measurements obtained in ambulatory patients.

In a subset of patients receiving LTOT, pre-LTOT and post-LTOT arterial blood gases were compared. Post-LTOT measurements were obtained opportunistically during subsequent hospital admissions for COPD exacerbations, as arterial blood gases are routinely reassessed prior to hospital discharge once patients reach clinical stability. Importantly, hospitalization rates were comparable between CaO_2_ groups, with a mean of 1.5±2.65 hospitalizations in patients with low CaO_2_ and 1.1±1.72 hospitalizations in those with normal CaO_2_, suggesting that the availability of post-LTOT measurements was driven by routine clinical practice rather than systematic differences in disease severity or study design. Consequently, hospitalization data were not used to define the baseline cohort but only to allow a longitudinal comparison of CaO_2_ before and after initiation of LTOT in a subset of patients.

**Figure 2:**
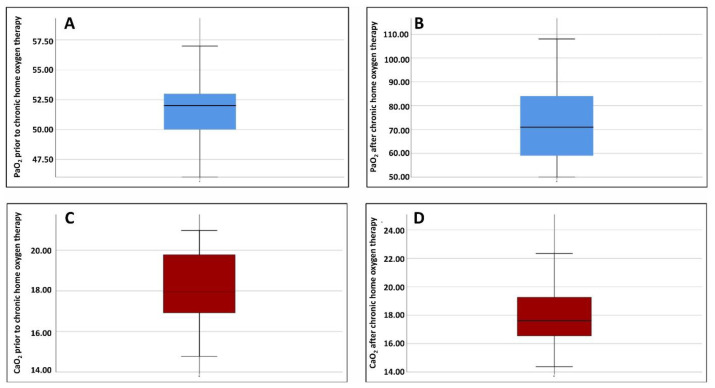
Changes in PaO_2_ and CaO_2_ after Initiation of Home Oxygen Therapy. After starting home oxygen therapy, a significant increase in arterial partial pressure of oxygen (PaO_2_) was observed **(A, B)**, while arterial oxygen content (CaO_2_) remained virtually unchanged **(C, D)**. This finding shows that, despite the improvement in PaO_2_, total oxygen transport capacity does not change substantially.

The LTOT was prescribed in 64 of 147 patients (43.8%). When stratified by CaO_2_ levels, 46.7% of patients with low CaO_2_, 40.2% of patients with normal CaO_2_, and none of patients with high CaO_2_ were receiving LTOT. The PaO_2_ increased significantly after LTOT, whereas CaO_2_ did not change significantly (Figure 2). Notably, 36.2% of patients maintained low CaO_2_ levels despite LTOT.

**Figure 3:**
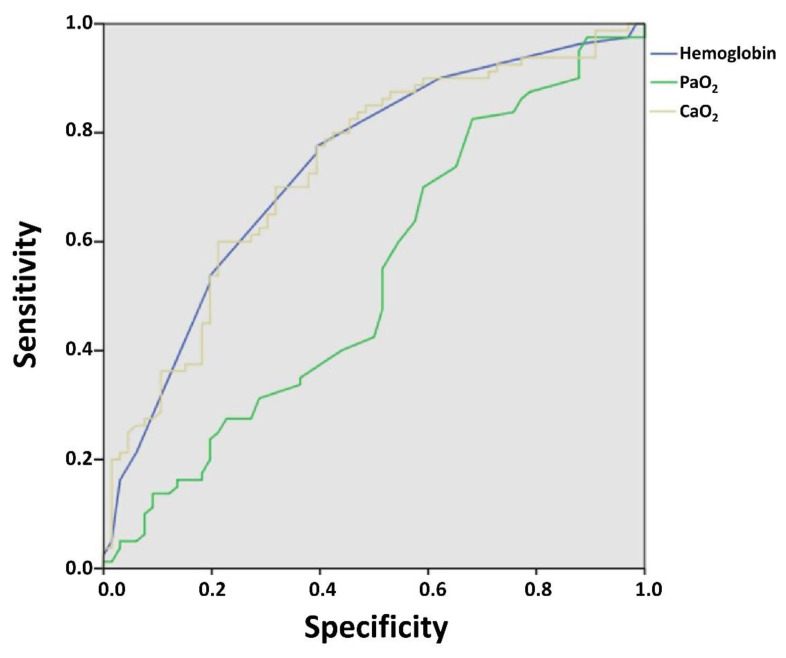
ROC Analysis Comparing Predictive Performance of Hb, CaO_2_, and PaO_2_ for Mortality in Patients with COPD. Hemoglobin and CaO_2_ showed similar discriminatory ability (AUC 0.735 and 0.732, respectively), whereas PaO_2_ demonstrated poor predictive performance (AUC 0.532), indicating limited ability to discriminate mortality risk. These findings suggest that markers reflecting oxygen transport capacity provide greater prognostic information than PaO_2_ alone. COPD, chronic obstructive pulmonary disease; CaO_2_, arterial oxygen content; Hb, hemoglobin; PaO_2_, arterial partial pressure of oxygen.

In an additional ROC analysis including hemoglobin alone, Hb showed a discriminatory ability for mortality comparable to that of CaO_2_, whereas PaO_2_ demonstrated poor predictive performance (Figure 3). These findings reinforce that markers reflecting oxygen transport capacity provide better prognostic discrimination than PaO_2_ alone. Although hemoglobin showed a similar discriminatory ability, CaO_2_ provides an integrated physiological measure combining hemoglobin concentration and arterial oxygenation, which may better reflect systemic oxygen delivery in COPD.

**Figure 4:**
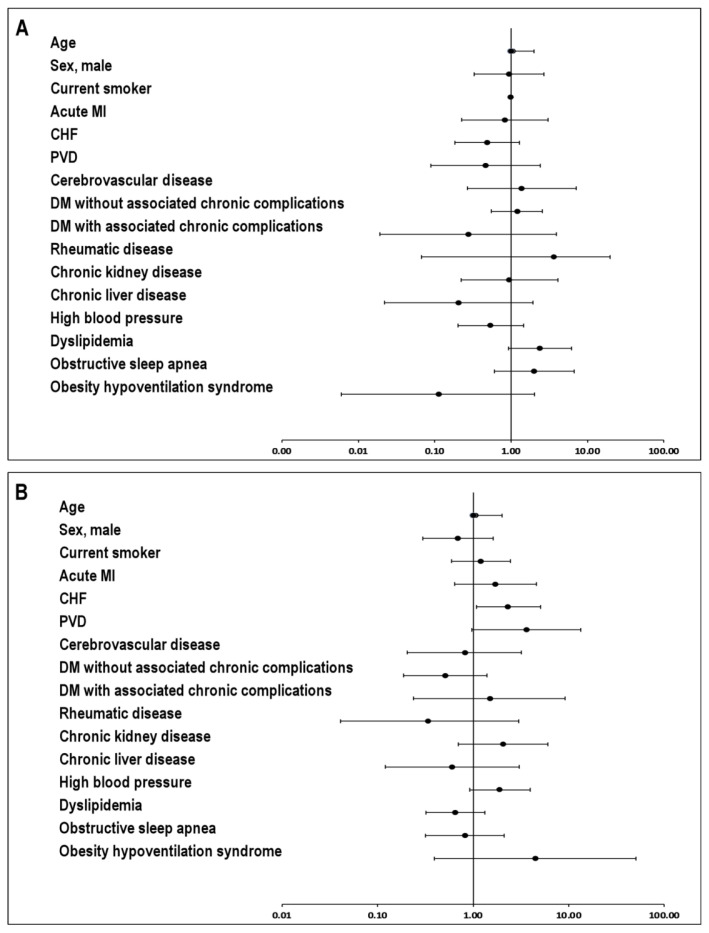
Odds Ratios for All-cause Mortality. Odds ratios (OR) for all-cause mortality with their respective 95% confidence intervals (95% CI) are shown for each variable included. **A: Univariate Analysis**. Only age was significantly associated with an increased risk of the outcome. **B: Multivariate Analysis**. Both age and chronic heart failure were found to be independent risk factors. CHF, chronic heart failure; DM, diabetes mellitus; MI, myocardial infarction; PVD, pulmonary vascular disease.

To further evaluate the prognostic performance of oxygenation parameters, a Cox proportional hazards regression analysis was performed including age, Hb, PaO_2_, and CaO_2_ (Figure 4). Age was independently associated with mortality (HR 1.04; 95% CI 1.01–1.07; *P*=0.002). In contrast, PaO_2_ was not significantly associated with mortality (HR 1.01; 95% CI 0.98–1.03; *P*=0.61). Moreover, hemoglobin was not independently associated with the outcome when included in the same model (HR 0.99; 95% CI 0.71–1.38; *P*=0.95). Arterial oxygen content (CaO_2_) showed a protective trend, although this did not reach statistical significance in the multivariable model (HR 0.82; 95% CI 0.63–1.08; *P*=0.16). These findings suggest that PaO_2_ alone provides limited prognostic information for mortality in COPD patients, whereas parameters reflecting systemic oxygen transport, such as CaO_2_, may better capture the complex physiological determinants of oxygen delivery.

## DISCUSSION

Our findings confirm that CaO_2_ provides better discrimination for mortality than PaO_2_. However, due to the modest specificity and predictive values observed, CaO_2_ should be interpreted as a complementary physiological marker rather than a standalone risk stratification tool. The low correlation between PaO_2_ and CaO_2_ highlights the limitations of PaO_2_ as an isolated marker of oxygenation. The cutoff point of 17.4 mL/dL appears clinically meaningful, with a high sensitivity (79%) for identifying patients at increased mortality risk. Nevertheless, due to its limited specificity, PPV, and NPV, CaO_2_ does not allow for complete risk stratification and must be interpreted in conjunction with other clinical and physiological markers. These observations reinforce the concept that indicators integrating oxygenation and oxygen transport, such as CaO_2_, offer a more meaningful clinical assessment than PaO_2_ alone in COPD. This may help identify patients who could potentially benefit from further evaluation for LTOT.

In our study, CaO_2_ was obtained from arterial blood gas analysis, which includes direct measurement of hemoglobin and arterial oxygen saturation, thus avoiding the inaccuracies associated with calculations based on pulse oximetry. This reinforces the prognostic validity of CaO_2_ compared to purely calculated estimates. Although the correlation between CaO_2_ and hemoglobin is physiologically predictable, our findings demonstrate that this physiological dependence is clinically relevant: CaO_2_, which integrates hemoglobin and oxygenation, demonstrated better discriminatory ability than PaO_2_, which only reflects dissolved oxygen.^2^–^4^ Therefore, two patients with identical hemoglobin levels may have markedly different CaO_2_ depending on their oxygenation status. The weak correlation between PaO_2_ and CaO_2_ observed in our cohort supports this concept. Although hemoglobin alone showed similar discriminatory ability in ROC analysis, CaO_2_ provides an integrated physiological measure combining hemoglobin concentration and arterial oxygenation.

Since CaO_2_ is mathematically derived from hemoglobin and oxygen saturation, including both variables in the same multivariable model would introduce collinearity. Therefore, CaO_2_ was used as the primary variable representing systemic oxygen transport. The risk of low CaO_2_ increases with age and in the presence of HFrEF. Although only age was significant in the univariate analysis, multivariate adjustment was performed to control for clinically relevant confounding factors. The emergence of CHF as an independent predictor indicates that its effect was masked in the univariate analysis by age and lung function. Aging causes loss of lung elasticity, increased dead space, and reduced circulating Hb, which decreases CaO_2_.^9^,^10^ In HFrEF, lower cardiac output compromises systemic oxygenation. In HFpEF, CaO_2_ may be normal at rest, but exercise-induced hypoxemia and reduced peripheral oxygen extraction during exertion have been documented. These differences could explain why in our study the risk of low CaO_2_ was higher in HFrEF than in HFpEF. Despite a significant improvement in PaO_2_ following the initiation of LTOT, CaO_2_ did not show any significant changes. Our data suggest that, even with LTOT, patients experiencing exacerbations are unable to achieve adequate CaO_2_ levels. This could imply that, even with normal PaO_2_ values, conventional oxygen therapy may be sufficient in the acute setting.

This discrepancy can be explained by the fact that CaO_2_ depends not only on arterial oxygenation, but also on Hb concentration and functionality, which are affected by chronic inflammation, anemia, COPD progression, advanced age, and comorbidities such as CHF,^9^,^10^ which may limit the overall response to LTOT. It is plausible that an improvement in PaO_2_ does not necessarily imply a greater effective supply of tissue oxygen.

### Limitations

The main limitations of this study include its retrospective and single-center design. In addition, the high CaO_2_ group was small, and residual confounding related to unmeasured clinical variables cannot be excluded. These factors may limit the generalizability of the results and the ability to establish causal relationships. However, our data underscore the clinical relevance of CaO_2_ as a potential prognostic marker in patients with COPD. Post-LTOT arterial blood gas measurements were available only in a subset of patients because follow-up measurements were obtained opportunistically during subsequent hospital admissions. Another potential consideration is that LTOT was prescribed according to established clinical criteria based primarily on PaO_2_ rather than CaO_2_.

Therefore, treatment decisions were not influenced by CaO_2_ levels. This reduces the likelihood that the observed association between CaO_2_ and mortality was driven by treatment allocation. In addition, the proportion of patients receiving LTOT was similar across CaO_2_ categories, suggesting that differences in outcomes were unlikely to be explained solely by variations in oxygen therapy prescription.

## CONCLUSIONS

Our findings indicate that CaO_2_ may provide a more physiologically meaningful measure of oxygen transport and mortality risk than PaO_2_ alone in patients with COPD. Prospective, multicenter studies are needed to validate these results and further investigate the prognostic value of CaO_2_.

## Abbreviations

CaO_2_: arterial oxygen content
COPD: chronic obstructive pulmonary disease
Hb: hemoglobin
HFpEF: heart failure and preserved ejection fraction
HFrEF: heart failure and reduced ejection fraction
LTOT: long-term oxygen therapy
PaO_2_: arterial partial pressure of oxygen
SatO_2_: arterial oxygen saturation
